# Out of sync: a Shared Mental Models perspective on policy implementation in healthcare

**DOI:** 10.1186/s12961-019-0499-x

**Published:** 2019-11-27

**Authors:** Jenna M. Evans, Karen S. Palmer, Adalsteinn D. Brown, Husayn Marani, Kirstie K. Russell, Danielle Martin, Noah M. Ivers

**Affiliations:** 1 DeGroote School of Business, McMaster University, 1280 Main Street West, Hamilton, Ontario Canada; 2 Women’s College Research Institute, Women’s College Hospital, Toronto, Ontario Canada; 3 Faculty of Health Sciences, Simon Fraser University, Burnaby, British Columbia Canada; 4 Institute of Health Policy, Management and Evaluation, Dalla Lana School of Public Health, University of Toronto, Toronto, Ontario Canada; 5 Dalla Lana School of Public Health, University of Toronto, Toronto, Ontario Canada; 6 Li Ka Shing Knowledge Institute, St. Michael’s Hospital, Toronto, Ontario Canada; 7 Institute for Health Systems Solutions and Virtual Care, Women’s College Hospital, Toronto, Ontario Canada; 8 Department of Family and Community Medicine, Women’s College Hospital and University of Toronto, Toronto, Ontario Canada

**Keywords:** policy implementation, shared mental models, frames, change management, health reform, activity-based funding

## Abstract

The impact of policy ambiguity on implementation is a perennial concern in policy circles. The degree of ambiguity of policy goals and the means to achieve them influences the likelihood that a policy will be uniformly understood and implemented across implementation sites. We argue that the application of institutional and organisational theories to policy implementation must be supplemented by a socio-cognitive lens in which stakeholders’ interpretations of policy are investigated and compared. We borrow the concept of ‘Shared Mental Models’ from the literature on industrial psychology to examine the microprocesses of policy implementation. Drawing from interviews with 45 key informants involved in the implementation of a hospital funding reform, known as Quality-Based Procedures in Ontario, Canada, we identify divergent mental models and explain how these divergences may have affected implementation and change management. We close with considerations for future research and practice.

## Introduction

Recent reviews demonstrate a growing interest in establishing the next frontier of research and practice on policy implementation [1–4]. An implementation perspective entails understanding how, why and by whom policy is put into effect and with what results [4]. The purpose of policy implementation research is to examine whether policies are implemented as conceived and to explain implementation success or failure [5, 6]. Congruent interpretations among stakeholders regarding policy goals and the means to achieve them are important for both implementation fidelity and policy achievement [4, 7]. We define congruence as the extent to which there is a shared understanding of formal policy goals and the means to achieve them between high level political officials and staff in organisations, where change occurs and its impact is intended to be felt. Congruence is challenging to achieve because policy initiatives tend to be political, ambiguous and involve multi-organisational and multi-stakeholder contingencies and conflicts [4, 7–11].

The study of congruence in policy implementation demands a socio-cognitive perspective in which stakeholders’ mental models – their abstractions of how a given policy works – are prioritised, investigated and compared. However, the existing literature on policy implementation contains few cognitive studies of stakeholders. Issues of cognition are often mentioned in the analysis and interpretation of existing papers on policy implementation, but rarely explicitly incorporated into the research design [4, 7].

A large body of work examines how stakeholders frame policy issues, but this literature focuses primarily on policy development or formulation as opposed to policy implementation [12]. Furthermore, scholars conducting research on policy tend to be situated in a sociological, not psychological, tradition [4]. Framing theory, for example, which focuses on “*underlying structures of belief, perception and appreciation*”, is a branch of institutional theory [13]. A Shared Mental Model (SMM) lens may provide new or complementary insights on the microprocesses of policy implementation.

In this essay, we argue for the application of SMM theory to the study of policy implementation. SMM theory, from the literature on industrial psychology, posits that a shared understanding of tasks and roles among interdependent stakeholders involved in an initiative maximises collective performance [14]. Empirical research over the past 20 years confirms a positive relationship between SMMs and performance at the team level [15, 16] and organisational level [17]. More recently, SMM theory has been extended to the inter-organisational- or health system-level to examine the implementation of integrated care interventions spanning multiple health and social service organisations [18–20]. Drawing from this line of inquiry, SMM theory may also help advance the study and practice of policy implementation in healthcare and beyond.

Below, we introduce the concept of SMMs. We then use the implementation of Quality-Based Procedures (QBPs) in hospitals in the province of Ontario, Canada, to illustrate how divergent mental models may have influenced the policy implementation process. This is followed by a discussion of contributions and limitations of a SMMs lens on policy implementation as well as directions for future research.

### Theoretical framework: Shared Mental Models and collective performance

Mental models are internal representations of external reality [21]. These small-scale models of how the world works consist of knowledge and beliefs that enable individuals to interpret situations and take action [21]. When multiple individuals develop a common psychological understanding of their environment or of an event, this is referred to as a SMM [14, 16]. SMMs allow individuals to develop a shared understanding of what is happening, what is likely to happen next and why it is happening, thus enabling behaviour that is consistent and coordinated in completing interdependent tasks [14, 16, 22]. Identical mental models are not necessary or, in many cases, feasible; rather the aim is a level of consensus that is broad enough to provide the common meaning needed for organised action while accommodating differing views or understandings on specific issues [23, 24]. Thus, we define ‘shared’ as ‘similar’ and ‘overlapping’, not ‘identical’ mental models.

SMM theory has been tested primarily in military, information technology and engineering contexts [16], and more recently in healthcare [25]. A meta-analysis of 65 studies shows SMMs positively predict team functioning and performance regardless of the context, team type or measures used [15]. Although SMMs are team-level phenomena, the extent to which mental models are shared (or not) can influence performance beyond the group level [17, 26, 27]. For example, in a study examining healthcare personnel’s mental models of clinical practice guidelines and organisational guideline implementation, personnel in high-performing facilities exhibited SMMs of guidelines while personnel in lower performing facilities did not [17]. SMM theory has also been extended to the inter-organisational or health system-level to examine the implementation of large-scale change involving multiple stakeholder groups [18–20].

We argue that policy implementation in healthcare is a prime example of large-scale multi-stakeholder change and would thus benefit from a SMM lens. Indeed, the policy literature often describes implementation problems resulting from divergent understandings or misunderstandings [7, 9, 10]. SMMs fall into three broad, inter-related categories task related, team related and beliefs [14, 24]. In the context of policy implementation, these three categories can be reframed as policy related, stakeholder related and beliefs, as defined in Table 1. The ‘beliefs’ category is akin to how ‘frames’ are defined by institutional theorists. Although diverse mental models may improve decision-making during the policy development stage, at the point of action or implementation, SMMs in these three areas (Table 1) may be required for success [7, 26].

**Table 1:** Types of shared mental models for policy implementation

Shared mental model type	Definition	Importance
Policy related	Shared knowledge and understanding of policy goals, implementation strategies, contingency plans and environmental conditions as well as shared policy-relevant knowledge	Stakeholders work towards a common vision efficiently and effectively
Stakeholder related	Shared knowledge and understanding of respective responsibilities, role interdependencies and communication mechanisms as well as shared understanding of others’ expertise, skills and preferences	Stakeholders accurately tailor their behaviour to what they expect from others, particularly when time and circumstances do not permit explicit communication and strategising
Beliefs	Shared perceptions, opinions and values of key issues relevant to the policy	Stakeholders interpret and frame issues in a similar way, thereby reducing conflict

Note: These mental model types and definitions are specific to policy implementation, and were adapted from the categories used in Shared Mental Model theory, namely task related, team related and beliefs [11, 21]

## Methods

### Context

In 2012, the Ontario Ministry of Health and Long-Term Care in Canada announced an ambitious policy aimed at improving healthcare delivery in the province – QBPs. QBPs represent the first major change in Ontario’s hospital funding mechanism since the introduction of global budgets in 1969 [28]. QBPs, a variant of activity-based funding [29], replace a small portion of each hospital’s annual lump sum global budget with a ‘patient-based’ funding approach. Under this QBP funding model, hospitals are paid a pre-set reimbursement rate for episodes of care based on diagnoses or procedures [10]. To guide the provision of care, each QBP has an implementation handbook that includes an evidence-based, best-practice clinical pathway.

### Data collection and analysis

In 2016, we undertook a qualitative evaluation of the implementation of QBPs using an embedded case study design, involving document review and semi-structured interviews with 45 key informants identified mainly through purposeful sampling [10, 30]. Participants were involved in designing the QBP policy (*n* = 12, ‘level one’ participants), in enabling adoption of QBPs across hospitals (*n* = 11, ‘level two’ participants), and in implementing QBPs within hospitals (*n* = 22, ‘level three’ participants). For hospital implementers (level three), we selected five hospitals from the initial 71 hospitals implementing QBPs across Ontario. We used a purposeful, stratified sampling approach based on a survey of executives from 16 provincial agencies. These executives were well-positioned to comment on hospital success or lack thereof with regards to QBPs. The five selected hospitals varied in size, geographic location, status as an academic versus community hospital and perceived relative success with QBPs [10]. Within these five hospitals, we interviewed four hospital leadership groups (chief executive, financial/decision support, clinical and medical) using purposeful sampling. Snowball sampling was pursued beyond these groups where appropriate.

Interviews were recorded, transcribed verbatim and coded inductively using Quirkos® qualitative data analysis software. Thematic analysis, incorporating framework analysis, was used to generate themes from the data. Additional details on QBPs and on the study’s recruitment, data collection and analytical approaches are provided elsewhere [10, 30]. Ethics approval was received from the Women’s College Research Institute Research Ethics Board (REB# 2016–0016-E).

### Thematic analysis results

The results of the qualitative evaluation described above suggest that QBP implementation was suboptimal [10]. QBPs were designed with a 3-year phase-in, with the intent to fund 30% of care by Fiscal Year 2014/15. Yet, as of Fiscal Year 2016/17, only about 15% of care was funded by QBPs, suggesting challenges with implementation. Participant comments from all three levels corroborate these challenges, as demonstrated by the sample quotes below:

“*I don’t know* [how it is going]*. I think some would say QBPs haven’t really lived up to their expectations. Others probably believe that it’s going really well. It all depends who you talk to*” (Level (L)1, Participant (P)8).

“[We are] *pushing back and saying slow down because this is tougher to manage than we thought, and it’s got all kinds of complications in the implementation … I think the execution needs to be improved for the whole QBP and health system funding reform process*” (L2, P13).

“*There has not been change management dollars behind change. Fundamentally, change actually costs to implement and then you get the benefits down the line*” (L3, P23).

The thematic analysis revealed that 4 years into implementation, confusion and misunderstandings of the primary goal of QBPs and the QBP funding mechanism persisted [10]. These differences in understanding of QBPs within and across stakeholder groups prompted the research team to revisit the coded interview data using a SMM lens. Select codes were reviewed to identify shared or divergent mental models using the categories in Table 1. Examples of the codes we reviewed include (1) ‘Purpose/goals of QBPs’, (2) ‘Mechanisms by which QBPs will achieve intended goal(s)’ (including the sub-code ‘Degree of shared understanding of how/why QBPs work’) and (3) ‘Evaluation of QBP implementation’ (including the sub-code ‘Degree of shared impression of implementation success/failure’).

### A Shared Mental Models lens on the implementation of Quality-Based Procedures in Ontario, Canada

We identified three key examples of divergent mental models in QBP implementation, each of which maps to a SMM type in Table 1. Below, we describe these divergent mental models and explain how they may have affected implementation.

The interviews revealed a lack of consistency and clarity over time in stakeholders’ understanding of the goals and the means by which those goals would be achieved [10], suggesting weak policy-related SMMs. “*A significant challenge in the implementation of QBPs*”, noted one participant, “*is that there has been no clarity as to what the primary purpose is*” (L1, P45). Some participants argued that QBPs primarily aimed “*to engage clinicians*” (L1, P4), “*focus on quality*” (L2, P12) or “*reduce practice variation*” (L1, P8), while others noted that “*QBPs are really more about funding than they are about quality*” (L3, P37). Reflecting on the policy as a whole, two participants involved in the implementation of QBPs in hospitals stated, “*We don’t really understand what QBPs are all about. Most of us don’t even know what the right questions are to ask*” (L3, P8) and “*I don’t think that everyone in the organisation has the same understanding of QBPs*” (L3, P7). Another participant suggested that “*we should have had a collective think about how to do it* [QBPs]*, and that did come later, but it came after the fact*” (L2, P12).

Differing perspectives of the definition and goal(s) of QBPs contributed to divergent views of who within hospitals should lead QBP implementation [10], suggesting weak stakeholder-related SMMs. Some participants, for example, argued that “*the CFO* [Chief Financial Officer] *plays an essential role. That’s where the leadership is for the most part for a QBP*” (L1, P8), while others “*made a deliberate decision for the implementation to be led at a clinical programme level, not by the finance team*” (L3, P43). One participant recounted a conversation among senior leaders regarding who the executive sponsor for QBPs should be within their hospital: “*A clinical VP* [Vice President] *was one of the nominees. We say, ‘it should be you because this is about quality of care’. He says, ‘No, QBPs are more about funding. It’s health system funding reform. It’s a financial thing. It should be led by our CFO.’ And our CFO was sitting on the other side of the table saying ‘No, this is about clinical quality improvement. Yes, it has a financial component, but this really has got to be owned by the clinical service areas’*” (L3, P44).

Finally, there appeared to be varied levels of trust in both the expressed promise of QBPs and in those overseeing and leading QBP implementation. These divergent beliefs resulted in some stakeholders feeling cynical about QBPs and others feeling optimistic. For example, one participant attributed provider non-compliance with QBP pathways at his site to an “*I don’t believe in it, I don’t have to do that*” attitude (L3, P42), while another participant described the opposite sentiment: “*They said ‘we’re going to implement the recommendations in these handbooks, because we trust that they’re the right thing. There are smart people on these expert panels. They seem to be based on evidence. So, we’re going to give it the benefit of the doubt’*” (L1, P14). Leader behaviours influenced trust, as this participant also recounted: “*They need to believe it too. Some of them come and sit on a committee and then they go off and bad mouth something and that’s all it takes for people to say ‘oh, man, the leaders don’t really believe in this’*” (L1, P8). A participant from an agency supporting QBP adoption noted that, “*People are completely open to using QBPs … But they need to have the confidence that the decisions being made are practical and responsible*” (L2, 20).

The examples above demonstrate the extent to which policy-related, stakeholder-related and belief-related mental models failed to converge across stakeholders. A participant involved in the design of QBPs articulated the challenge of congruence nicely: “*It’s one thing to do this work at the provincial level, but unless those signals get transmitted clearly to the local level, then you’re going to have a breakdown in how it gets understood and what the response to that signal is*” (L1, P4).

## Discussion

As the QBP example demonstrates, how policies are understood by stakeholders shapes how they are ultimately enacted across implementation sites [7, 31]. This finding is supported by previous literature on policy implementation in healthcare [9, 10, 20]. What differentiates our paper from others is the novel application of SMM theory to this particular implementation problem. We argue that a SMM lens on policy implementation may enhance our understanding of how stakeholders operationalise policies and help explain implementation success or failure. The complex nature of healthcare systems generally, and the implementation of complicated policies like QBPs specifically, highlights the importance of SMMs to effective policy implementation. Furthermore, policies are often highly couched to avoid conflict, but this also reduces opportunities to identify underlying “*problems of understanding*” [27] and build SMMs; hence, the need for explicit attention to SMMs throughout the policy implementation process.

Existing literature on policy implementation recognises the importance of stakeholder cognitions [4, 7], but rarely directly examines and compares them. For example, Sabatier’s Advocacy Coalition Model [32] has an individual cognitive component, but it focuses on how stakeholders seek out like-minded stakeholders to form coalitions. SMM theory focuses our attention on establishing common ground across such coalitions and identifies what kinds of knowledge and beliefs need to be shared to support coordinated action. A SMM lens may also improve understanding of what differentiates ‘early adopters’ from ‘late adopters’ and ‘laggards’. These labels, from Diffusion of Innovation Theory [33], are often used to describe stakeholders’ receptivity (or lack thereof) to new policies (e.g. [34, 35]), but with little consideration for their cognitive foundations. Comparing the mental model content and congruence of early and late adopters over time may generate new knowledge on facilitating policy implementation. Addressing these socio-psychological and behavioural gaps in the literature on policy implementation will require multi-disciplinary research teams, including psychologists [4], and longitudinal qualitative or mixed methods designs.

The SMM concept is embedded in a well-established theoretical and empirical body of work in industrial psychology [15, 16]; drawing from this literature may support and enhance the rich insights garnered from the application of framing theory in public policy generally, and healthcare policy specifically. SMMs may build on the concept of frames in three ways: in terms of content, stage of the public policy process, and future directions for research and practice. With regards to content, SMMs go beyond a focus on belief structures (or ‘logics’) – where institutional theories, such as framing theory, tend to focus – to include an emphasis on knowledge structures. Knowledge of a change (i.e. what the change is, how it will be implemented, what impact(s) it will have) has been linked to less resistance to change [36]. With regards to stage of the policy process, SMMs are inherently focused on the minutiae of implementation rather than solely on the beliefs and values underlying the early stages of problem identification and policy formulation. Finally, the literature on SMMs identifies several variables and recommendations that may offer novel directions for scholars and practitioners. For example, a growing body of empirical research examines interventions to facilitate development of SMMs, including training, planning, feedback and reflexivity exercises in which mental models are surfaced and discussed [16]. In particular, pre-briefing or planning behaviours before or during a team task have been linked to SMM development and enhanced team performance; these behaviours include setting goals and prioritising tasks, discussing how constraints will be managed and the consequences of errors, exchanging preferences or expectations, determining what types of information all team members have access to and what types of information are held by only certain members, and clarifying roles, sequencing and timing [22]. Scenario planning – a process of visualising what future events are probable, what their consequences would be, and how to respond to them – has also been identified as a means to change individual mental models and build collective SMMs [37, 38].

How might have the implementation of QBPs differed if a SMM lens had been considered and prioritised? There would have been an expectation and appreciation that mental models will diverge across diverse stakeholder groups. As a result, forums and tools might have been developed to assess mental models regularly; for example, regular surveying to gauge understanding or cross-role and cross-institutional meetings in which a checklist of SMMs guides the discussion. These early assessments may have resulted in earlier identification of divergent mental models and potentially more targeted strategies to support QBP adoption. The primary adoption support designed to enable implementation was a handbook for each funded diagnosis and/or procedure. Yet, evidence on the implementation of QBPs shows that these handbooks did not address specific barriers to change nor hospitals’ capacity to manage change [30]. Had there been appreciation for the incongruences across stakeholders regarding QBPs, including understanding of the goals of QBPs, who within hospitals should lead implementation, and whether QBPs could deliver as promised, adoption supports might have been designed differently to address these barriers.

## Conclusion

We argue that the concept of SMM from the literature on industrial psychology may have theoretical and practical utility in efforts to improve policy implementation. Given the original focus of SMM theory on teamwork, SMM theory may be most appropriate for application to policy implementation when (1) coordinated action is required among diverse stakeholders and (2) the relevant stakeholders are easily identified and limited in number. SMM theory may also be particularly useful in cases of top-down policy implementation, which is the typical route for implementation in highly regulated quasi-market systems [39]. Future research should examine the relevance and utility of a SMM lens on policy implementation.

## Data Availability

Request for access to study data should be directed to KP or NI, or to the Research Ethics Board at Women’s College Hospital Research Ethics Office. De-identified datasets generated and/or analysed during the current study are available from the corresponding author on reasonable request and upon approval from the abovementioned Research Ethics Board. Any release of data will require a new ethics approval.

## Abbreviations

QBPs: quality-based procedures
SMMs: shared mental models
